# Verzögerte Lokalreaktion mit subkutaner Infiltration nach Impfung mit mRNA-1273 – ein bisher unbeschriebenes Reaktionsmuster eines COVID-Arms

**DOI:** 10.1007/s00105-022-04986-7

**Published:** 2022-03-31

**Authors:** Lukas Kofler, Stephan Forchhammer

**Affiliations:** Universitäts-Hautklinik, Eberhard-Karls Universität Tübingen, Liebermeisterstr. 25, 72076 Tübingen, Deutschland

**Keywords:** Coronavirus, mRNA-Impfstoff, T‑Zellen, Immmundermatologie, Lymphozytäres Infiltrat, Coronavirus, mRNA vaccine, T cells, Immunodermatology, Lymphocytic infiltrate

## Abstract

Der Impfstoff mRNA-1273 gegen SARS-CoV‑2 wurde Anfang 2021 in Europa zugelassen. Inzwischen liegen einige Fallberichte zu verzögerten Lokalreaktionen nach Impfung vor („COVID-Arm“). Es wurden dabei oberflächliche lymphozytäre Infiltrate beschrieben, jedoch keine Beteiligung der tiefen Dermis oder Subkutis. Wir berichten über den Fall eines gesunden 32-jährigen Mannes mit Beteiligung der tiefen Dermis und Subkutis nach Impfung mit mRNA-1273. Dieser Fall zeigt erstmals eine verzögerte T‑Zell-vermittelte Reaktion mit tiefem Reaktionsmuster, wobei sich das dermale perivaskuläre und periadnexiale Infiltrat von der papillären Dermis bis in die tiefe retikuläre Dermis und die Subkutis erstreckte. Es zeigte sich ein überwiegend lymphozytäres Infiltrat mit einer Beimischung von Histiozyten und neutrophilen Granulozyten, vereinzelten Mastzellen und spärlichen eosinophilen Granulozyten.

## Abstract (en)

The mRNA-1273 vaccine against SARS-CoV‑2 was approved in Europe in early 2021. Meanwhile, there are a number of case reports of delayed local reactions after vaccination (“COVID arm”). In these reports, superficial lymphocytic infiltrates were described, but no involvement of the deep dermis or subcutis. We report the case of a healthy 32-year-old man with involvement of the deep dermis and subcutis after vaccination with mRNA-1273. This case is the first to show a delayed T‑cell mediated reaction with a deep pattern of reaction, with the dermal perivascular and periadnexal infiltrate extending from the papillary dermis into the deep reticular dermis and subcutis. The infiltrate was predominantly lymphocytic with an admixture of histiocytes and neutrophil granulocytes, scattered mast cells and sparse eosinophil granulocytes.

## Anamnese und klinischer Befund

**Figure 1:**
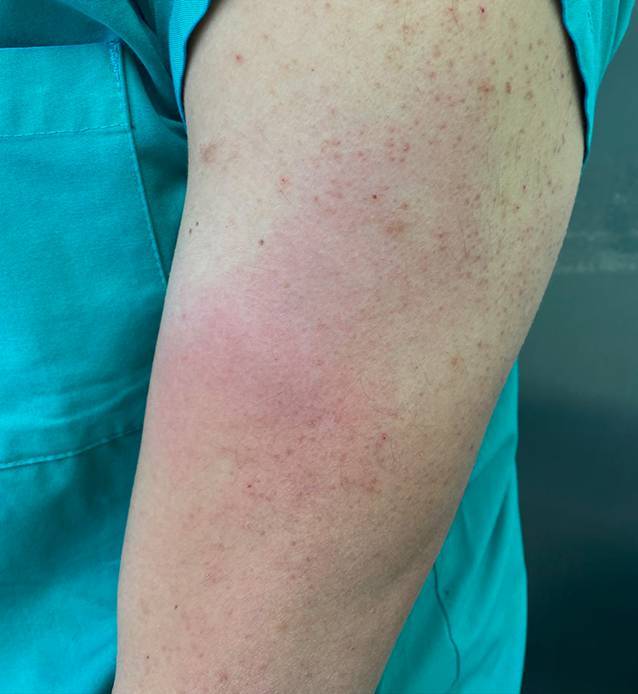

**Figure 2:**
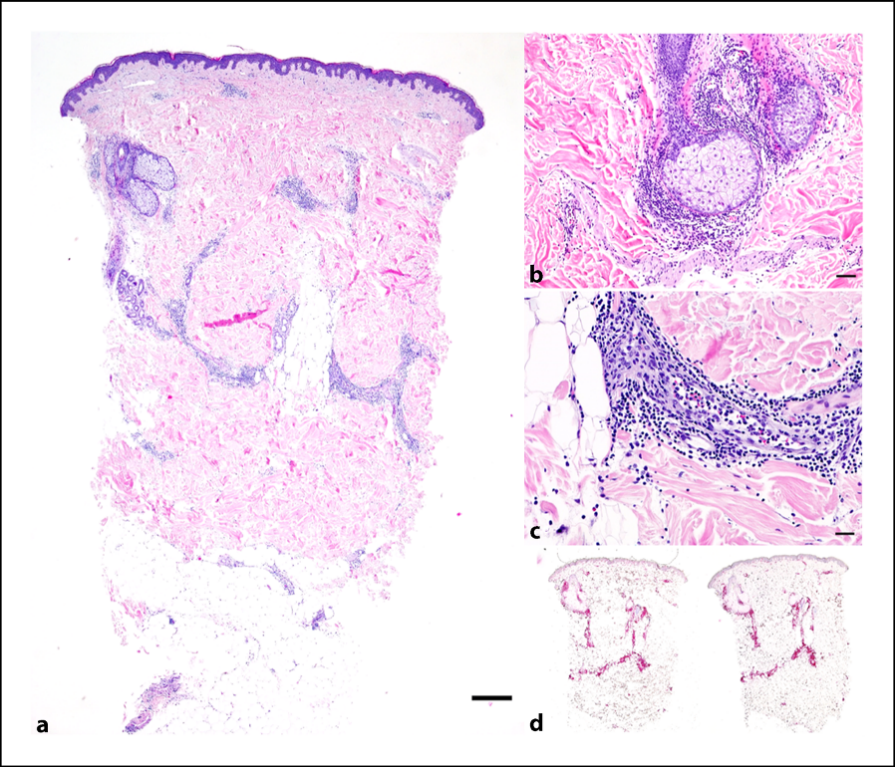

Wir stellen den Fall eines ansonsten gesunden 32-jährigen Mannes vor, der mit mRNA-1273 erstgeimpft wurde und die Impfung ohne anfängliche lokale Reaktion vertrug. Anamnestisch lagen weder Allergien (insbesondere nicht auf Impfstoffe, Kosmetika oder Darmspüllösungen) noch Hinweise auf vorangegangene Impfreaktionen vor. Zehn Tage nach der Impfung wurde in unmittelbarer Nähe der Injektionsstelle eine erythematöse Plaque mit anfänglich urtikariellem Aussehen beobachtet, die schließlich einen Durchmesser von mehr als 10 cm erreichte (Abb. 1). Nach der zentrifugalen Ausbreitung verblasste die Plaque zentral und bildete sich anschließend innerhalb von 24 h ohne systemische oder topische Behandlung vollständig zurück. Eine Stanzbiopsie aus dem Zentrum der Plaque zeigte eine dermale, perivaskuläre und periadnexiale Dermatitis (Abb. 2a, b). Die Epidermis war von orthokeratotischem Horn bedeckt ohne Anzeichen von Spongiosa oder Interface-Dermatitis. Das dermale perivaskuläre und periadnexiale Infiltrat erstreckte sich von der papillären Dermis bis in die tiefe retikuläre Dermis und die Subkutis. Es handelte sich hauptsächlich um ein lymphozytäres Infiltrat mit einer Beimischung von Histiozyten und neutrophilen Granulozyten, vereinzelten Mastzellen und spärlichen eosinophilen Granulozyten (Abb. 2c). Die Hautgefäße waren mit neutrophilen Granulozyten gefüllt. Es wurden keine vaskulitischen oder vaskulopathischen Veränderungen festgestellt. Die Immunhistochemie zeigte überwiegend CD3^+^-T-Zellen mit einer ausgewogenen Expression von CD4^+^ und CD8^+^ (Abb. 2d, e). Die CD20-Färbung zeigte nur ein geringes B‑Zell-Infiltrat. Dieses histologische Muster kann bei verschiedenen Hauterkrankungen auftreten, darunter polymorphe Lichteruption, Lupus erythematodes, Morphea, Infektionskrankheiten (z. B. Lepra, Lues, *Borrelia burgdorferi*) und Arzneimittelreaktionen. Da das histologische Muster an einen Lupus erythematodes tumidus erinnert, wurden eine immunhistochemische Färbung mit CD123-Antikörper sowie eine Toluidin-Färbung durchgeführt. Es gab keine Vermehrung plasmazytoider dendritischer Zellen und keine dermalen Muzinablagerungen. CD138-Antikörper zeigten keine Hinweise auf eine Plasmazellpopulation im Infiltrat, die den Verdacht auf eine Infektionskrankheit, insbesondere auf ein Erythema chronicum migrans, erwecken könnte.

## Diagnose

Aufgrund der Klinik sowie der Histologie stellten wir die Diagnose einer Impfreaktion nach mRNA-basiertem Impfstoff („COVID-Arm“) mit Beteiligung der tiefen Dermis und Subkutis.

## Diskussion

Der mRNA-basierte Impfstoff mRNA-1273 gegen SARS-CoV‑2 wurde im Januar 2021 in Europa zugelassen. In einer Phase-3-Studie mit 30.420 Probanden wurden bei weniger als 1 % der Probanden verzögerte Reaktionen an der Injektionsstelle beobachtet [1]. Zu diesen Reaktionen gehörten ein verhärtetes Erythem und Empfindlichkeit bei Berührung an der Injektionsstelle. Blumenthal et al. berichteten über 12 Patienten mit ähnlichen Reaktionen 4 bis 11 Tage nach der Impfung [2]. Der hier vorgestellte Patient tolerierte die zweite Impfung ohne weitere lokale Reaktion, was gegen eine Sensibilisierung vom Typ IV spricht und mit Beobachtungen in der Literatur übereinstimmt [2].

Es hat sich gezeigt, dass die intramuskuläre Injektion zu einer (gewünschten) verlängerten Proteinexpression an der Injektionsstelle führt [3–5]. Nachdem der mRNA-Impfstoff an der Injektionsstelle von Antigen-präsentierenden Zellen internalisiert wurde, wird das Antigen von diesen Zellen in seiner nativen Form (oder als bereits prozessierte Peptide) über MHC‑I auf CD8^+^-Zellen oder als prozessierte extrazelluläre Antigene über MHC-II auf CD4^+^-Zellen präsentiert. Dadurch werden zytotoxische T‑Zellen aktiviert, und die Differenzierung von B‑Zellen durch T‑Helferzellen wird unterstützt [6, 7]. Wir beobachteten eine lokale Reaktion auf mRNA-1273 mit einer 10 Tage nach Injektion erstmals aufgetretenen und sich im Verlauf rasch ausbreitenden erythematösen Plaque an der Injektionsstelle und einer perivaskulären sowie periadnexialen oberflächlichen und tiefen lymphozytären Dermatitis als histologisches Korrelat.

Hautveränderungen nach einer COVID-19-Infektion sind gut bekannt [8]. Das histologische Muster, das bei Chilblain-ähnlichen, akralen Läsionen („COVID-Toes“) beschrieben wird, weist eine gewisse Ähnlichkeit mit unserem Fall auf mit einer oberflächlichen und tiefen perivaskulären und periadnexialen lymphozytären Entzündung, allerdings sind in unserem Fall keine keratinozytären Schäden und vaskulitischen Veränderungen vorhanden [9].

In einer neueren Studie wurden ähnliche histologische Befunde bei einem Patienten beschrieben, der nach einer Impfung gegen SARS-CoV‑2 eine große lokale Reaktion zeigte. In Übereinstimmung mit unserem Befund zeigte dieses Präparat oberflächliche perivaskuläre und perifollikuläre lymphozytäre Infiltrate, jedoch wurde keine Beteiligung im Bereich der tiefen dermalen Anteile beobachtet [2]. Wir berichten daher über die erste verzögerte T‑Zell-vermittelte Reaktion auf den neuartigen mRNA-basierten Impfstoff mRNA-1273 mit einem tiefen Reaktionsmuster, das bis in das subkutane Gewebe reicht.

## Fazit für die Praxis

- Sowohl im Rahmen einer COVID-19-Infektion als auch nach Impfungen wurden verschiedene Hautveränderungen beschrieben.
- Nach Impfungen gegen das SARS-CoV-2-Virus mit mRNA-basierten Impfstoffen wurden verzögerte Lokalreaktionen beschrieben. Diese sind in der Regel durch oberflächliche lymphozytäre Infiltrate gekennzeichnet. Dieser Fall zeigt erstmals auch eine bis in die Subkutis reichende, überwiegend lymphozytäre, T‑Zell-dominante Infiltration.
- Bei einer verzögerten Lokalreaktion ohne systemische Beteiligung oder Hinweis auf eine allergische Reaktion besteht kein erhöhtes Risiko einer schweren allergischen Impfreaktion bei Folgeimpfungen, worüber PatientInnen aufgeklärt werden sollten.

## References

[1] Baden LR, El Sahly HM, Essink B (2021). Efficacy and safety of the mRNA-1273 SARS-CoV-2 Vaccine. N Engl J Med.

[2] Blumenthal KG, Freeman EE, Saff RR (2021). Delayed large local reactions to mRNA-1273 Vaccine against SARS-CoV‑2. N Engl J Med.

[3] Pardi N, Hogan MJ, Porter FW, Weissman D (2018). mRNA vaccines—a new era in vaccinology. Nat Rev Drug Discov.

[4] Iavarone C, O’hagan DT, Yu D (2017). Mechanism of action of mRNA-based vaccines. Expert Rev Vaccines.

[5] Zhang C, Maruggi G, Shan H, Li J (2019). Advances in mRNA vaccines for infectious diseases. Front Immunol.

[6] Rauch S, Jasny E, Schmidt KE, Petsch B (2018). New vaccine technologies to combat outbreak situations. Front Immunol.

[7] Liang F, Lindgren G, Lin A (2017). Efficient targeting and activation of antigen-presenting cells in vivo after modified mRNA vaccine administration in rhesus macaques. Mol Ther.

[8] Freeman EE, McMahon DE, Lipoff JB (2020). The spectrum of COVID-19-associated dermatologic manifestations: An international registry of 716 patients from 31 countries. J Am Acad Dermatol.

[9] Kanitakis J, Lesort C, Danset M, Jullien D (2020). Chilblain-like acral lesions during the COVID-19 pandemic (“COVID toes”): histologic, immunofluorescence, and immunohistochemical study of 17 cases. J Am Acad Dermatol.

